# Haplotype-resolved telomere-to-telomere genome assembly of the dikaryotic fungus pathogen *Rhizoctonia cerealis*

**DOI:** 10.1038/s41597-025-05260-w

**Published:** 2025-06-06

**Authors:** Jiang-Na Han, Yu Li, Wei Li, Hao-Hao Yan, Fengping Yuan, Huai-Gu Chen, De-Jun Han, Zhen-Sheng Kang, Qing-Dong Zeng

**Affiliations:** 1 State Key Laboratory for Crop Stress Resistance and High-Efficiency Production, College of Plant Protection, Northwest A&F University, Yangling, 712100 Shaanxi China; 2 State Key Laboratory for Crop Stress Resistance and High-Efficiency Production, College of Agronomy, Northwest A&F University, Yangling, 712100 Shaanxi China; 3 Institute of Plant Protection, Jiangsu Academy of Agricultural Sciences, Nanjing, Jiangsu 210014 China

**Keywords:** Fungal genomics, Fungal biology

## Abstract

*Rhizoctonia cerealis*, the causal agent of sharp eyespot, is a highly destructive pathogen of wheat. Despite its global importance, the genetic and molecular mechanisms underlying virulence of *R. cerealis* remain poorly understood. *R. cerealis* is a dikaryotic organism and the haplotype phase has been isolated. Based on the PacBio HiFi, Oxford Nanopore, and Hi-C platforms, we assembled the first high quality telomere-to-telomere (T2T) haplotype-resolved genome of *R. cerealis*, with sizes of 41.50 and 41.05 Mb, and N50 sizes of 2.67 and 2.42 Mb, respectively. High consensus quality values of 57.75 and 57.09 for the two haplotypes validated the accuracy of the assembly. The assembly achieved R-AQI and S-AQI scores of 92.5 and 100, respectively, both indicating reference-level quality. A total 25,353 protein coding genes were predicted for the two haplotypes with a BUSCO score of 96.7%. The genome assembly will serve as the foundation for further research on allele-specific expression, genetic variation and evolution of *R. cerealis*.

## Background & Summary

*Rhizoctonia cerealis* Van der Hoeven (current teleomorph name: *Ceratobasidium cereale* Murray & Burpee, within the Basidiomycota phylum), a member of the *Rhizoctonia* anastomosis group AG-D, subgroup I (AG-DI), is a soilborne basidiomycete that causes sharp eyespot in wheat. This pathogen is widespread in temperate wheat growing regions of Europe, North America, Africa, Oceania, and Asia^1^. Sharp eyespot has been reported every year in China since 2016, with the affected area approaching 8 million hectares (https://www.natesc.org.cn/). Severe infection leads to death of young tillers, lodging, and appearance of white heads^1^. The number of resistant varieties among those currently grown is limited, and control strategies primarily depend on the use of fungicides. The identification of pathogenicity genes could provide crucial insights for disease control strategies. However, previous studies were hindered by lack of a high-quality reference genome sequence, which would be beneficial for comprehending the genetic basis of pathogenicity, biochemical mechanisms, and infection processes.

**Fig. 1:**
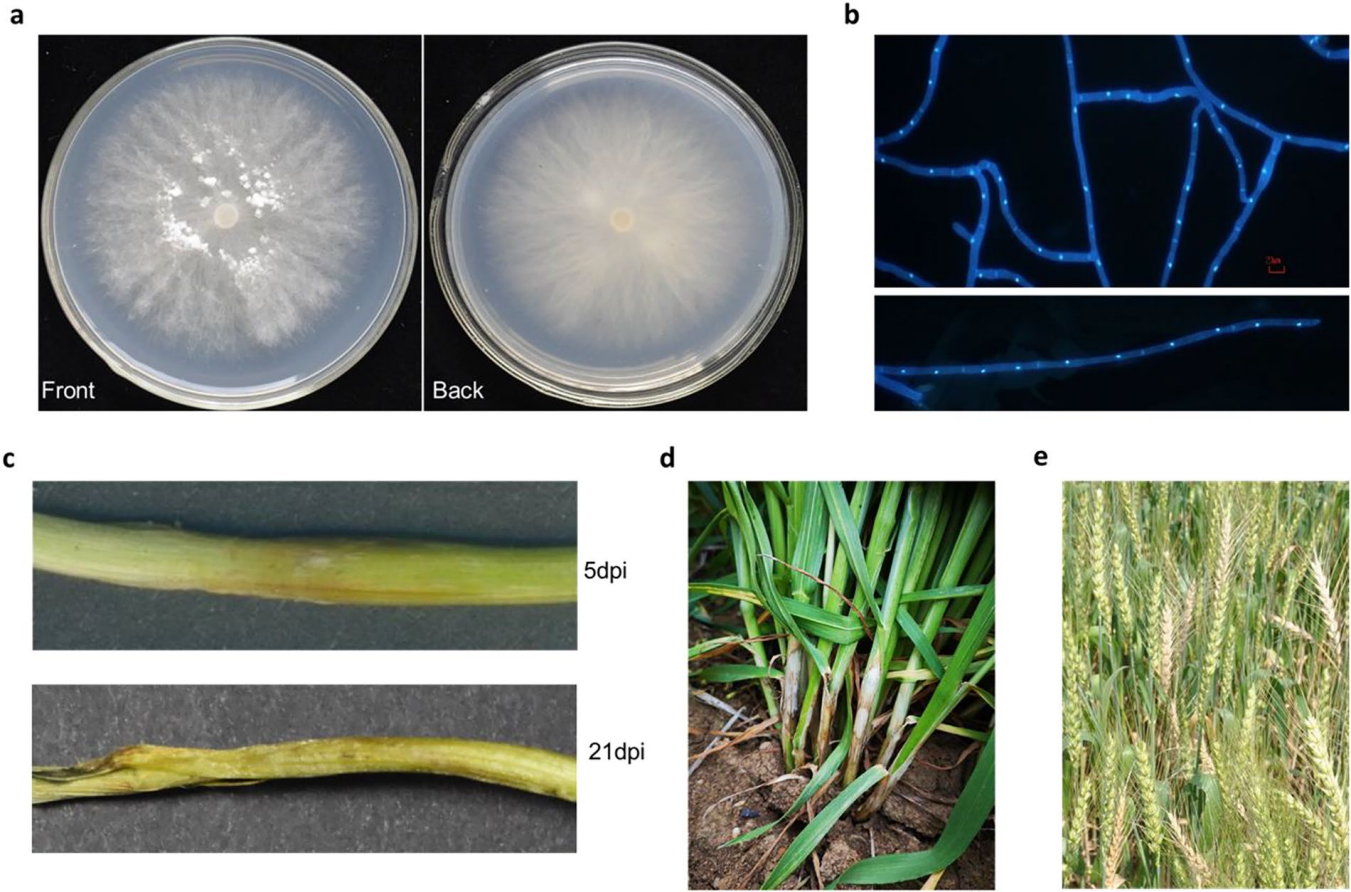
Morphology of *R. cerealis* and signs and symptoms of sharp eyespot in wheat. (**a**) The front and back sides of *R. cerealis* hyphae on a PDA agar plate at five days post inoculation. (**b**) All hyphal cells contain two nuclei as shown by DAPI staining. (**c**) Symptoms on wheat seedling leaf sheaths and stems at 5 dpi. (**d**) Field Symptoms on wheat leaf sheaths and stems in the field. (**e**) Symptoms on wheat spikes in the field.

*R. cerealis* is a dikaryotic fungus; its mycelium is white in color on a PDA agar plate (Fig. 1a), and each hyphal cell contains two nuclei (Fig. 1b). Over time, the inoculation, or infection, site on the wheat seedling leaf sheath and underlying stem changes from white to brown or black (Fig. 1c). Symptoms on wheat leaf sheaths and stems in the field appear as well-defined semicircular or oval lesions with gray-brown centers and a brown periphery (Fig. 1d), hence the name, eyespot. With prolonged infection spikes of infected tillers undergo premature ripening expressed as white heads (Fig. 1e). Constructing a haplotype-resolved genome facilitates identification of genomic variations between homologous chromosomes, such as single nucleotide polymorphisms (SNP) and structureal variations (SV). This enables exploration allele-specific gene expression at different stages of disease development, and elucidation of the impact of dikaryotism on the genome biology.

**Fig. 2:**
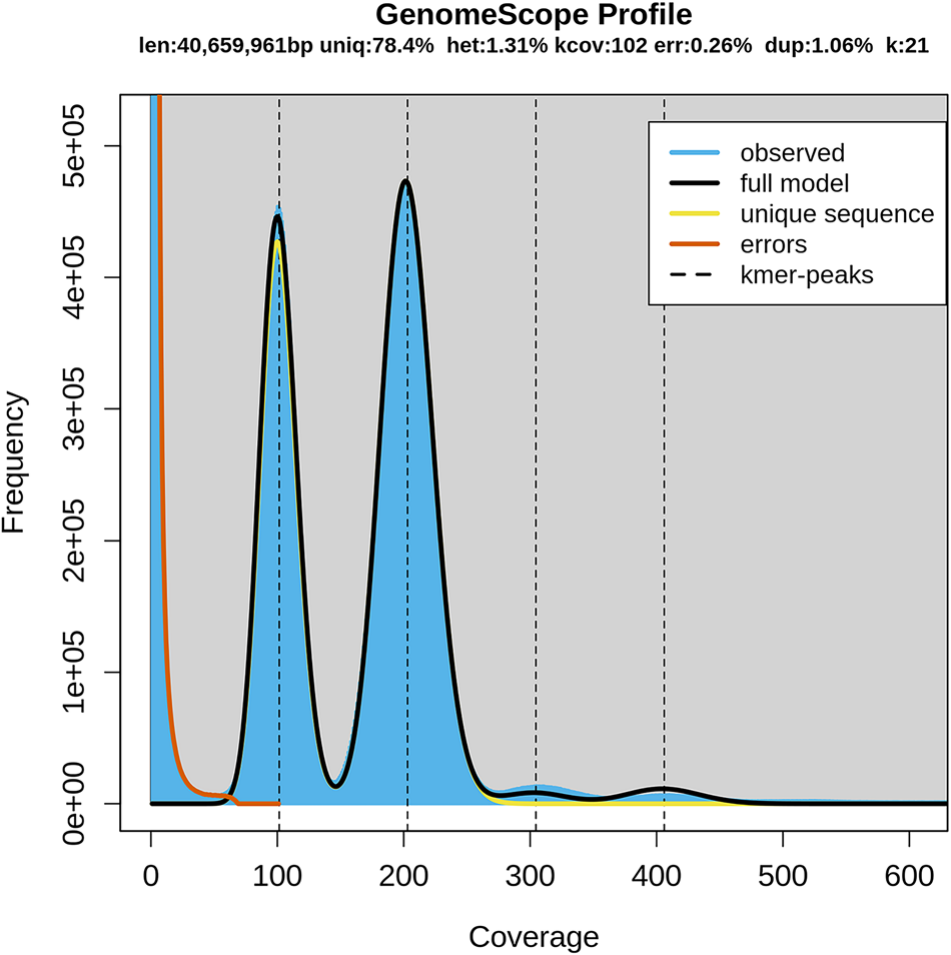
Genome size estimation based on 21-mer distribution. The size of the R0301 genome was estimated to be 40.66 Mb using the frequency distribution of 21-mer and the 1:2 depth ratio between peaks supports a diploid genome with 1.31% heterozygosity.

**Fig. 3:**
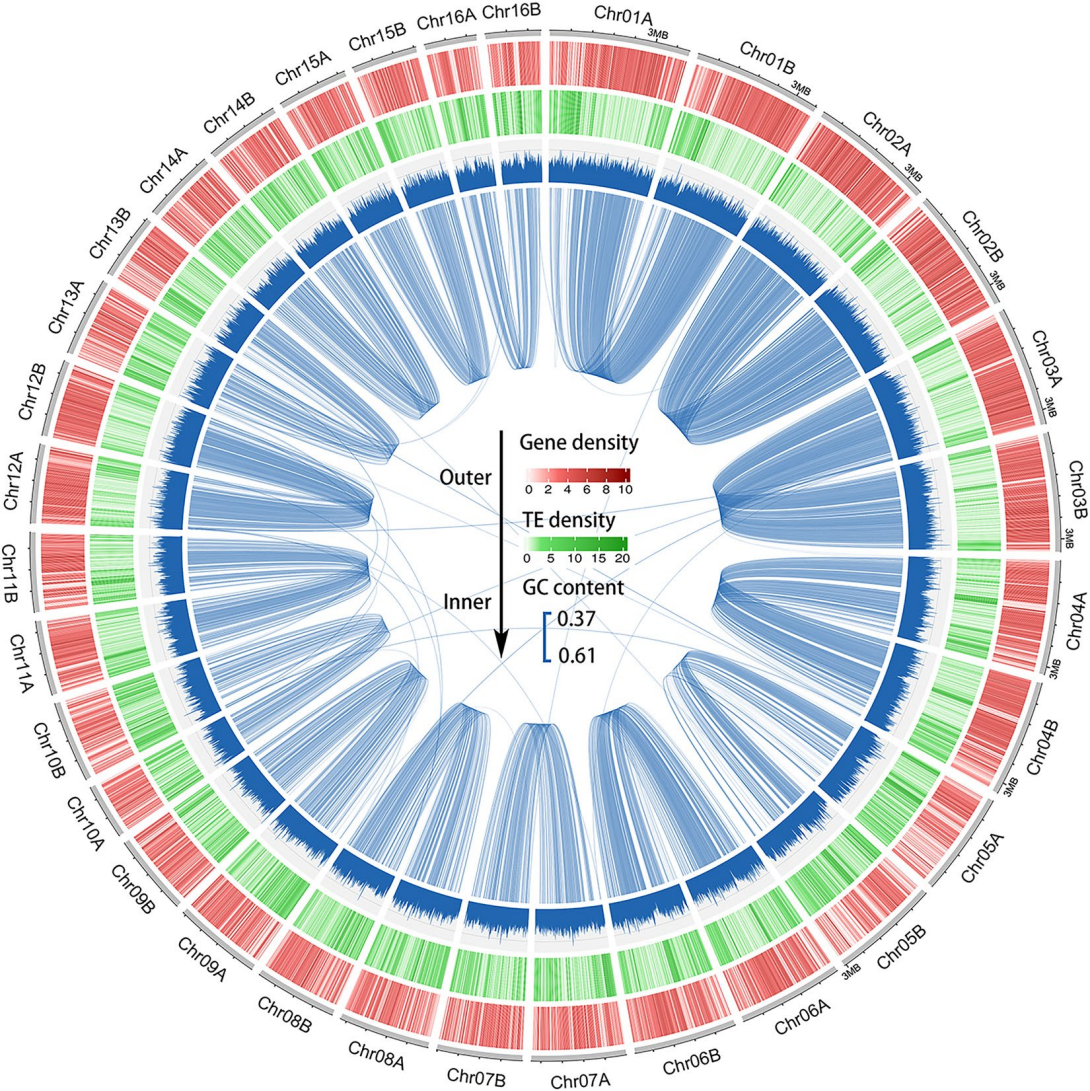
Circos plot illustrating the genomic features of isolate R0301 in 10 kb windows. Gene density, transposable element (TE) density, GC content, and collinear blocks between the two haplotype genomes (from the outermost to the innermost circles).

Until now, a chromosome-level genome assembly for *R. cerealis* has not been reported. Multiple sequencing platforms were used to obtain the present haplotype-resolved, chromosome-level genome assembly of strain R0301. Illumina sequencing yielded 10.65 Gp of clean data (Table 1), which was used for k-mer analysis. The haploid genome of R0301 was estimated to be 40.66 Mb, with a heterozygosity of 1.31% based on a k-mer value of 21 (Fig. 2). The PacBio sequel II platform generated 6.53 Gp of HiFi reads, with 99.9% accuracy and an N50 length of 19,881 bp (Table 1). The Nanopore ONT platform applying ultra-long sequencing technology, yielded an N50 length of 54,315 bp and 16.86 Gp of reads (Table 1), which were highly beneficial for assembling highly repetitive regions, such as centromeres and telomeres, and for generating a gap-free genome. High-throughput chromosome conformation capture (Hi-C) technology provided 17.83 Gb of reads for long-range scaffolding (Table 1). To acquire the telomere-to-telomere (T2T) and haplotype-resolved genome, HiFiasm^2^ (v.0.19.9) was employed along with the HiFi, ONT, and Hi-C data. Finally, the two haplotypes were separated, and the mitochondrion assembled. Based on Hi-C links, 41.50 Mb of contigs from the A haplotype genome and 41.05 Mb from the B haplotype genome were anchored onto chromosomes (Table 2). The final assembly consisted of 16 chromosome pairs without gaps, achieving a scaffold N50 of 2.67 Mb for haplotype A and 2.42 Mb for haplotype B (Fig. 3, Table 2). Gene annotation on the two haploid genomes identified 12,351 and 12,309 genes for the A and B genomes, respectively (Fig. 3, Table 3). Multiple assessment methods confirmed high continuity, base accuracy, and completeness of the haplotype-resolved assemblies (Table 2). The results of this study will be a useful resource for community research on pathogenicity, genetic variation, and evolution of *R. cerealis*.

**Table 1:** Summary of sequencing results for the isolate R0301 genome.

Library	Clean bases (Gb)	Clean reads	N50 length (bp)	Coverage (×)
PacBio HiFi	6.53	333,723	19,881	160.54
Oxford Nanopore	16.86	580,251	54,315	414.62
Illumina NovaSeq	10.65	35,496,183	2 × 150	261.90
Hi-C	17.83	59,444,893	2 × 150	438.60

**Note:** The estimated genome size (based on 21-mer analysis) was used for sequencing depth estimation.

**Table 2:** Summary statistics of HapA, HapB and their combined assembly.

Feature	Haplotype A	Haplotype B	Both haplotypes
No. of contigs	16	16	33
No. of bases	41,500,454	41,053,716	82,554,170
No. of chromosome gaps	0	0	0
Max	3,974,049	3,631,962	3,974,049
N50	2,670,066	2,418,312	2,670,066
No. of T2T chromosomes	7	12	19
Telomere number	22	28	50
GC (%)	48.84	48.78	48.81
Mito length	—	—	156,349
Quality (QV)	57.75	57.09	57.41
Completeness	80.53	80.49	99.42
R-AQI	92.58	92.50	92.54
S-AQI	100	100	100
Genome BUSCO (%)	98.9	98.7	99.1
Protein BUSCO (%)	94.0	94.3	96.7
LTR	33.60	28.75	28.69

**Table 3:** Statistics of gene model properties in the isolate R0301 genome.

Characteristic	HapA	HapB	Both haplotypes
Number of genes	12,351	12,309	24,660
Number of mRNAs	12,553	12,800	25,353
Number of CDSs	12,553	12,800	25,353
Number of exons	79,943	83,423	163,366
Mean gene length (bp)	1,755.2	1,813.3	1,784.2
Mean mRNA length (bp)	1,770.0	1,842.0	1806.4
Mean CDS length (bp)	1,376.8	1,382.4	1,379.6
Mean exons length (bp)	228.1	232.6	230.4

## Methods

### DNA extraction, library construction and sequencing

The CTAB^3^ method was used for extracting DNA used in Illumina sequencing. Sequencing was performed with an Illumina Novaseq instrument to obtain a PE 150-bp PCR-free library with a 350 bp insert size. High molecular weight (HMW) DNA was extracted following a protocol for BAC library construction^4^ and then sheared using a Covaris g-TUBE device with 20 kb settings. The sheared DNA was purified and concentrated with AmpureXP beads and subsequently used for single-molecule real-time (SMRT) bell preparation according to the manufacturer’s protocol. HMW DNA, also used for the 1D library, was prepared and sequenced by a Nanopore PromethION sequencer. The same nuclear DNA for Hi-C library construction was cross-linked and then digested with restriction enzyme DpnII, resulting in pairs of distally located but physically interacting DNA molecules. The sticky ends of these digested fragments were biotinylated and ligated to form chimeric circles. Biotinylated circles, chimeras of the physically associated DNA molecules from the cross-linking, were enriched, sheared, and sequenced with the Illumina Novaseq platform.

### RNA extraction, library construction and sequencing

RNA was extracted separately from *R. cerealis* cultured on potato dextrose agar (PDA) medium and from fungal-infected wheat sheaths at 7, 14, and 21 days post-infection (dpi). The extracted RNA was subsequently sequenced using the Illumina Novaseq platform.

### Genome size and estimation of heterozygosity

Based on the Illumina reads, Jellyfish (v2.2.10, -m 21)^5^ was used to count k-mers, and Genomescope (v1.0.0)^6^ was employed to determine genome size and the heterozygosity level.

### Haplotype-resolved genome assembly

HiFi reads, ultra-long reads, and Hi-C reads were assembled into contigs using hifiasm^2^ (v0.19.9) with parameters -h1 -h2 -ul for haplotype phasing. Clean Hi-C paired-end reads were aligned with the assembly using Juicer (v1.6)^7^ with the BWA^8^ algorithm to obtain the interaction matrix. The 3d-DNA (v201008) pipeline^9^ was applied to reorder the contigs into scaffolds. The position of the contigs was manually adjusted based on the Hi-C heatmaps visualized using JuicerBox^10^ (v1.11.08). The haplotype-resolved genome was polished using short reads and HiFi reads by NextPolish2^11^ with one iterative round.

### Gene prediction and functional annotation of the *R. cerealis* genome assembly

The Funannotate pipeline (v1.7.4, https://github.com/nextgenusfs/funannotate) was used for genome annotation. Seven steps were executed: (1) Renaming the fasta headers, (2) Sorting scaffolds by length to avoid downstream issues, (3) Soft-masking simple repeats in the genome with tantan, (4) Training gene models with the novaseq and Pacbio RNA-seq data trinity de novo assembly sequences, (5) Performing funannotate prediction with corresponding options after training, (6) Updating the annotation with UTR data and correcting gene models inconsistent with RNA-seq data, and (7) Annotating, which automatically incorporating the interproscan, antismash, phobius results.

Effectors play a crucial role in virulence of plant pathogenic fungi^12^. A comprehensive pipeline was employed to identify candidate effectors. Firstly, SignalP (v5.0)^13^ and Phobius (v1.01)^14^ were used to predict proteins with signal peptides. The proteins predicted by both sofrwares were combined and input into the next step. Then, TMHMM (v2.0c, to remove proteins with ExpAA larger than 18)^15^, ApoplastP (v1.0.1)^16^, LOCALIZER (v1.0.4)^17^, and EffectorP (v2.0)^18^ were used to predict candidate effectors.

AlleleFinder (https://github.com/sc-zhang/AlleleFinder) was used to identify alleles. Functional gene enrichment analysis was performed using the clusterProfiler package^19^. GO terms and KEGG pathways with p-adjust < 0.05 and q-value < 0.05 were regarded as significantly enriched. Proteins from the two haplotype genomes of R0301 were compared using BLASTP (-evalue 1e-6 -outfmt 6). Collinear blocks were identified using MCScanX (v0.8)^20^.

**Fig. 4:**
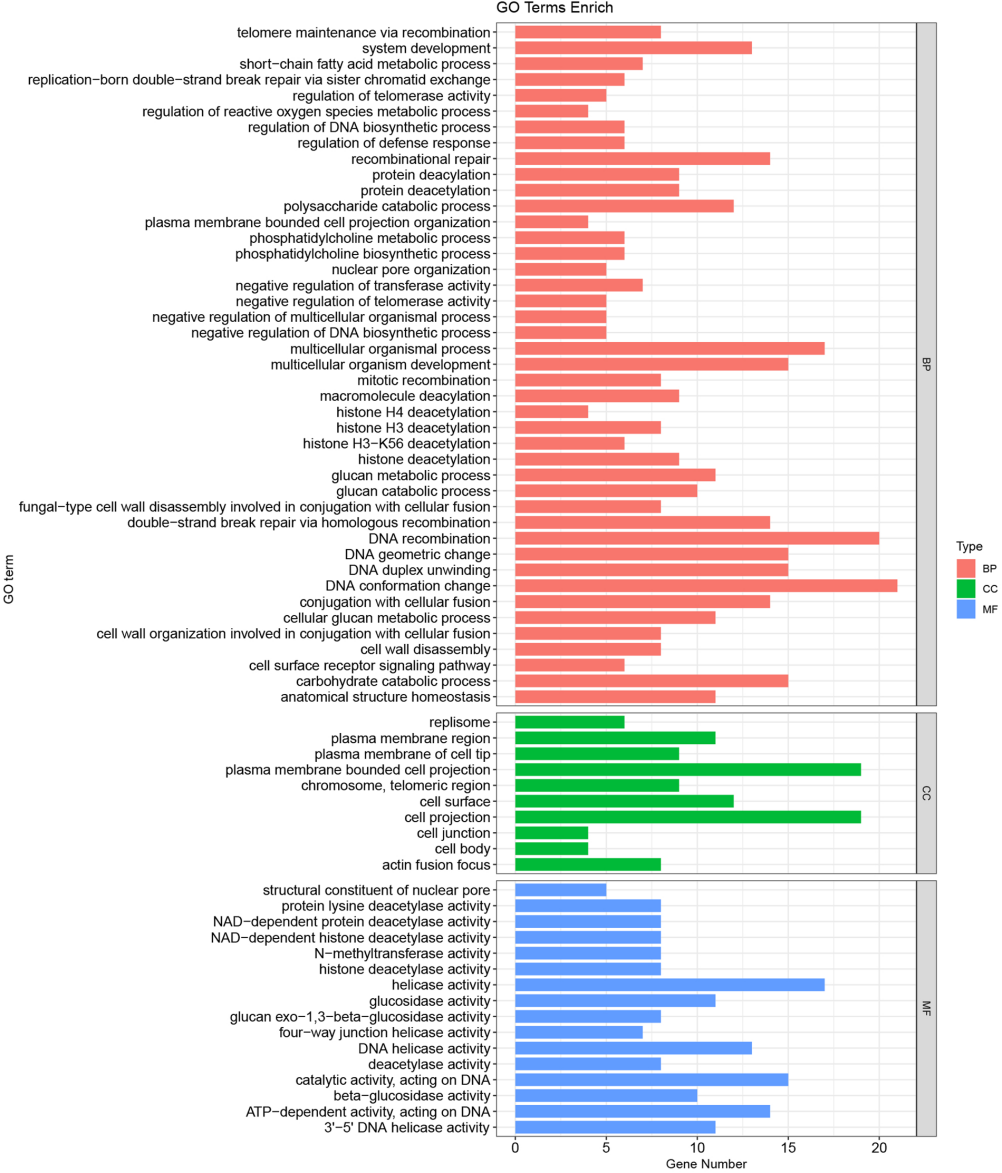
Gene ontology (GO) enrichment analysis of isolate R0301 genome specific genes. Significantly enriched terms (p.adjust < 0.05, q-value < 0.05) are categorized into three ontologies: biological processes (BP), cellular components (CC), and molecular functions (MF).

The Funannotate pipeline predicted 24,660 genes (25,353 isoforms). The properties of these gene, such as average CDS length and number of CDS in haplotypes A and B are presented in Table 3. Chromosome 02 (Chr02) exhibited the highest gene density (with an average 0.37 genes per kb), and Chr05 had the lowest gene density (0.20 genes per kb) (Table 4). Among tranlated proteins, 1,781 (7.02%) were annotated as secreted proteins (SPs), and 485 (1.91%) were annotated as cytoplasmic/apoplastic effectors (Supplementary Table S1). A majority of genes (81.3%, 10,582 of 13,130) were bi-allelic, and 18.7% (2,548 of 13,132) were mono-allelic, with 1,732 specifically in haplotype A and 816 in haplotype B (Table 4). Subsequent analysis revealed a significant influence on glucan metabolism and cell surface receptor signaling pathways (Fig. 4, Supplementary Table S2).

**Table 4:** Statistics of gene density, telomere length and allelic distribution in the isolate R0301 genome.

Chr	Length	Left TeloLen	Right TeloLen	Gene Num	Allele	Biallelic	Monoallelic	Gene Num/kb
Chr01A	3974049	105	95	1299	1281	1104	177	0.3326
Chr01B	3631962	0	95	1231	1153	1104	49	
Chr02A	3537565	75	75	1319	1310	1231	79	0.3727
Chr02B	3518529	100	95	1311	1256	1231	25	
Chr03A	3410335	100	0	1261	1249	1165	84	0.3704
Chr03B	3365530	105	0	1249	1206	1165	41	
Chr04A	3321515	0	105	1050	1048	945	103	0.3274
Chr04B	3224198	85	110	1093	1017	945	72	
Chr05A	3051069	0	100	612	617	464	153	0.2015
Chr05B	2988920	90	0	605	543	464	79	
Chr06A	2944841	0	90	854	854	717	137	0.2902
Chr06B	2915649	95	90	847	787	717	70	
Chr07A	2670066	0	0	727	727	566	161	0.2716
Chr07B	2579338	80	85	699	617	566	51	
Chr08A	2494804	90	85	660	662	524	138	0.2644
Chr08B	2418312	90	95	639	575	524	51	
Chr09A	2339498	95	90	669	657	516	141	0.2823
Chr09B	2301716	95	85	641	583	516	67	
Chr10A	2298281	100	70	398	439	379	60	0.1991
Chr10B	2253158	80	85	508	448	379	69	
Chr11A	2234249	100	0	640	646	572	74	0.2885
Chr11B	2233565	95	0	649	603	572	31	
Chr12A	2176744	0	65	779	756	691	65	0.3618
Chr12B	2135415	95	85	781	733	691	42	
Chr13A	2113121	90	0	559	522	408	114	0.2527
Chr13B	2065663	85	110	497	452	408	44	
Chr14A	2003024	95	105	571	579	499	80	0.2989
Chr14B	1880905	95	90	590	543	499	44	
Chr15A	1858721	105	105	591	584	473	111	0.3356
Chr15B	1571006	105	80	560	517	473	44	
Chr16A	1567676	95	0	362	383	328	55	0.2534
Chr16B	1474746	90	90	409	365	328	37	
Total		HapA		12,351	12,314	10,582	1,732	0.2941
		HapB		12,309	11,398	10,582	816	0.3035

Note: Allele enumeration excludes paralogous genes, resulting in a lower allele count compared to total gene number.

Transposable elements (TEs) were detected and annotated by HiTE^21^; 17.78% of the genome consisted of TEs. Long terminal repeats (LTRs) and non-LTR retrotransposons were the most abundant TEs, accounting for 14.34% of the two haplotype genomes (Table 5).

**Table 5:** Transposable elements found in the genome of isolate R0301.

Class	Count	Masked (bp)	Masked (%)
LTR	9,272	11,318,167	13.71
SINEs	84	14821	0.02
Penelop	0	0	0
LINEs	729	501,593	0.61
DNA transposons	4,912	2,381,033	2.88
Rolling-circles	623	340,066	0.41
Unclassified	422	120,233	0.15

### Synteny analysis

**Fig. 5:**
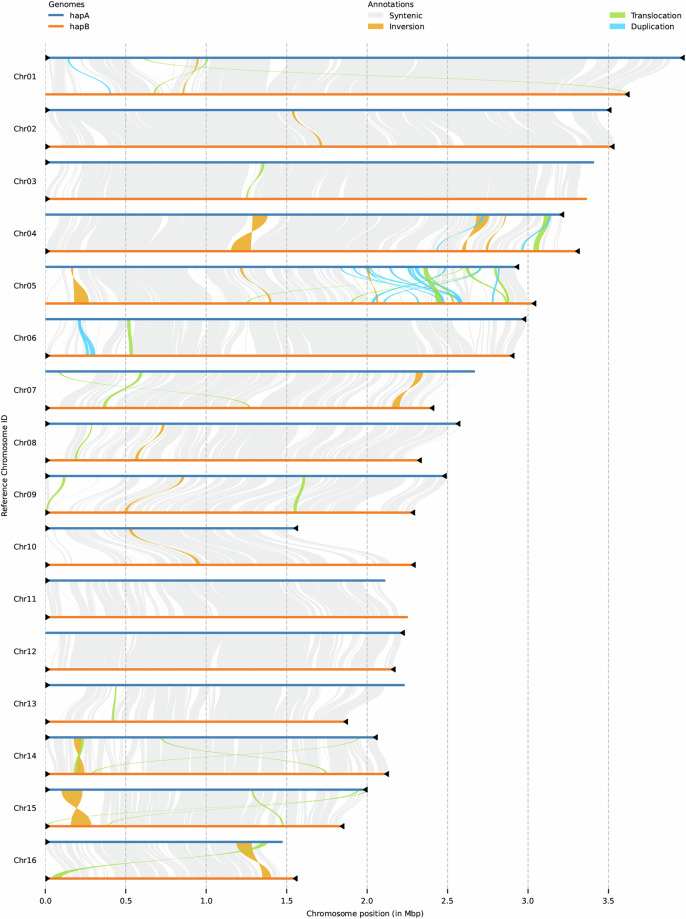
Comparative genomic landscape of synteny, structural variation, and telomere distribution in the haplotype-resolved genome. The 16 chromosomes belonging to HapA and HapB are shown in dark blue and orange, respectively. Synteny blocks between homoeologous regions are shown in grey. The black triangles indicate the presence of telomere sequence repeats.

MUMmer^22^ was used for whole-genome alignments with the default parameters to investigate the differences between the two haplotypes. The alignment results were filtered using delta-filter with the parameters ‘-m -i 80 -l 100’. After format conversion with the ‘show-coords’ command, SyRI (v1.6.3)^23^ was employed to detect syntenic regions and structural variations using the default parameters. Plotsr (v1.1.1)^24^ was used to visualize these variations. A total of 1,443 syntenic regions with a cumulative size of 58.89 Mb (71.34%) was detected (Fig. 5), indicating moderate similarity between the two haplotypes. A total of 427,567 SNPs and 22,409 indels (11,686 insertions and 11,073 deletions) were identified between HapA and HapB. Additionally, 1,240 translocations with a cumulative size of 6.77 Mb (~8.20%), 31 inversions with a cumulative size of 1.36 Mb (~1.64%), and 3,640 duplications with a cumulative size of 8.94 Mb (~10.82%) were detected (Fig. 5).

## Data Records

The PacBio HiFi and assemblies of *R. cerealis* isolate R0301 generated in this study are available in NCBI under BioProject PRJNA1187694 and PRJNA118790. The Oxford Nanopore, Hi-C, Illumina sequencing reads of R0301 were obtained from the sequencing data deposited in the BioProject PRJNA717151. The PacBio HiFi sequencing reads of R0301 have been deposited in the NCBI Sequence Read Archive database with accession group numbers SRP546896^25^. The Oxford Nanopore, Hi-C, Illumina sequencing reads of R0301 have been submitted to the NCBI Sequence Read Archive database (SRP312258^26^). Genome assembly is available from GenBank in the NCBI with accession numbers GCA_049000075.1^27^ and GCA_048999985.1^28^. The genome assembly and gene annotation results were also deposited in the Figshare database^29^.

## Technical Validation

### Evaluation of the assembled genome

**Fig. 6:**
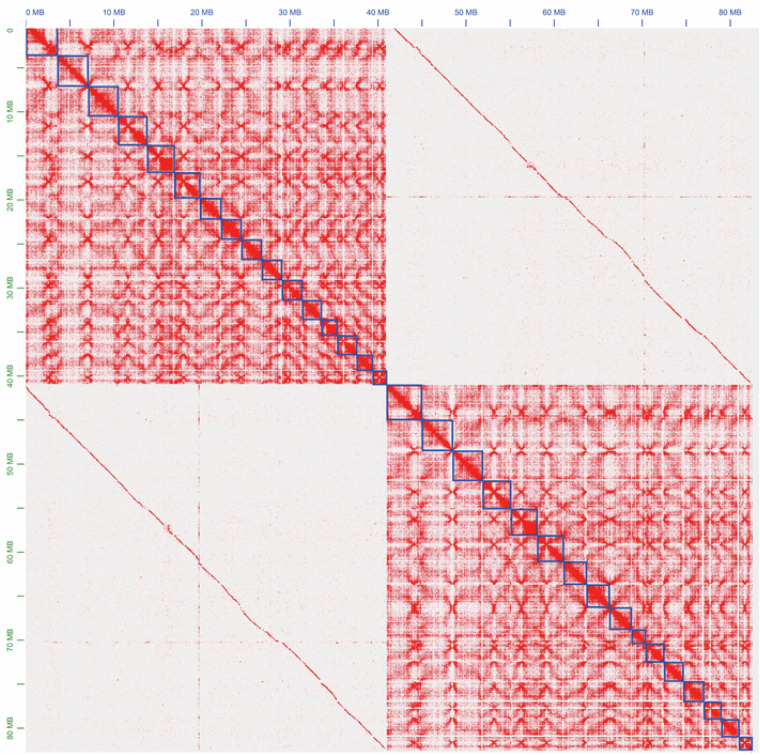
Inter-chromosomal Hi-C contact map of the full dikaryotic genome assembly for isolate R0301 (32 chromosomes). The nuclear HapA and HapB display a clear signal of spatial separation, the blue boxes represent chromosomal regions.

The quality of the genome assembly was evaluated using multiple methods. (1) Accuracy of the Hi-C based chromosome construction was evaluated by visualizing the chromatin contact matrix and contact maps using JuicerBox (v1.11.08)^10^. The Hi-C heatmap showed no errors in the two haplotype genomes (Fig. 6). (2) The completeness and duplication score of the haplotype A and B genomes were evaluated by BUSCO (v5.7.1)^30^ with the “basidiomycota odb9” database. The complete BUSCO scores (including single-copy and duplicated) of the two haplotypes as well as their combined assembly, accounted for 98.9%, 98.7% and 99.1% in genome mode (Table 2), respectively, suggesting good completeness of the genome assembly. (3) The accuracy of the genome was evaluated by mapping the WGS sequencing reads against the genome and calculating the mapping rate and coverage by Qualimap2^31^ and Pandepth^32^. The mapping rate was over 93.3%, and the coverage rate (>1X) was 100% for all three data types in both the HapA and HapB genomes (Supplementary Table S3). (4) CRAQ^33^ was used to evaluate the assembly errors of the R0301 genome. It used clipping information by mapping the original sequencing reads againest the *de novo* genome assembly, defining a reference level Assembly Quality Indicator (AQI over 92.50%) (Table 2). (5) Merqury (v1.1)^34^ was used to assess k-mer completeness and the consensus quality value of the R0301 genome assemblies based on the 21-mer hybrid Merqury kmer database of Illumina PCR-free reads. The HapA and HapB genomes, as well as their combined assembly, had a quality values (QV) of 57.75, 57.09 and 57.41, respectively (Table 2). (6) The LTR Assembly Index (LAI)^35^ value method was used to evaluate the assembly level of the genome based on repeat sequences. The LAI values for the genoems were 33.60, 28.75 and 28.69 (Table 2). (7) A total of 50 telomeres (Fig. 5; Table 2; Table 4) were identified across 32 chromosomes in the haplotype-resolved, gap-free genome of R0301 according to the characteristic repetitive base sequence of telomeric regions (CCCTAAA/TTTAGGG). Complete T2T assemblies were achieved for 19 chromosomes, with 7 T2T assemblies for haplotype A and 12 for haplotype B (Fig. 5; Table 2). These results indicate that the R0301 assembly constitutes the first haplotype-resolved, chromosome-scale genome resource for the *R. cerealis* species, reaching a near-complete standard with high contiguity and base accuracy.

### Evaluation of the gene annotation

The quality of the predicted proteins was assessed using BUSCO (v5.7.1)^30^ with the “basidiomycota odb9” database in protein mode. The complete BUSCO scores for the two haplotypes, as well as their combined assembly, were 94.0%, 94.3% and 96.7%, respectively (Table 2). These results indicated a high level of quality in the gene annotation process.

## Supplementary information

Supplementary Table S1, Supplementary Table S2, Supplementary Table S3

## Data Availability

All software and pipelines employed in this study were executed with the parameters detailed in the Methods section. In cases where specific parameters for the software were not provided, default parameters recommended by the developer were utilized. Circos visualizations were generated using ShinyCircos-V2.0^36^.

## References

[1] Hamada, M. S., Yin, Y. N., Chen, H. G. & Ma, Z. H. The escalating threat of *Rhizoctonia cerealis*, the causal agent of sharp eyespot in wheat. *Pest Manag. Sci.* **67**, 1411–1419 (2011). 21726039 10.1002/ps.2236

[2] Cheng, H. Y. *et al*. Haplotype-resolved assembly of diploid genomes without parental data. *Nat. Biotechnol.* **40**, 1332–1335 (2022). 35332338 10.1038/s41587-022-01261-x PMC9464699

[3] Rogstad, S. H. Saturated nacl-ctab solution as a means of field preservation of leaves for DNA analyses. *Taxon* **41**, 701–708 (1992).

[4] Zeng, Q. D. *et al*. Construction and characterization of a bacterial artificial chromosome library for the hexaploid wheat line 92R137. *Biomed Res. Int.* **2014**, 9 (2014). 10.1155/2014/845806 PMC4026951 24895618

[5] Marcais, G. & Kingsford, C. A fast, lock-free approach for efficient parallel counting of occurrences of k-mers. *Bioinformatics* **27**, 764–770 (2011). 21217122 10.1093/bioinformatics/btr011 PMC3051319

[6] Vurture, G. W. *et al*. GenomeScope: fast reference-free genome profiling from short reads. *Bioinformatics* **33**, 2202–2204 (2017). 28369201 10.1093/bioinformatics/btx153 PMC5870704

[7] Durand, N. C. *et al*. Juicer provides a one-click system for analyzing loop-resolution Hi-C experiments. *Cell Syst.* **3**, 95–98 (2016). 27467249 10.1016/j.cels.2016.07.002 PMC5846465

[8] Li, H. & Durbin, R. Fast and accurate short read alignment with Burrows-Wheeler transform. *Bioinformatics* **25**, 1754–1760 (2009). 19451168 10.1093/bioinformatics/btp324 PMC2705234

[9] Dudchenko, O. *et al*. De novo assembly of the *Aedes aegypti* genome using Hi-C yields chromosome-length scaffolds. *Science* **356**, 92–95 (2017). 28336562 10.1126/science.aal3327 PMC5635820

[10] Robinson, J. T. *et al*. Juicebox.js provides a cloud-based visualization system for Hi-C data. *Cell Syst.* **6**, 256–258 (2018). 29428417 10.1016/j.cels.2018.01.001 PMC6047755

[11] Hu, J. *et al*. NextPolish2: a repeat-aware polishing tool for genomes assembled using HiFi long reads. *Genom. Proteomics Bioinformatics* **22**, 6 (2024). 10.1093/gpbjnl/qzad009 PMC12016036 38862426

[12] Bai, X. *et al*. A candidate effector protein PstCFEM1 contributes to virulence of stripe rust fungus and impairs wheat immunity. *Stress Biology* **2**, 21 (2022). 37676523 10.1007/s44154-022-00042-5 PMC10441960

[13] Almagro Armenteros, J. J. *et al*. SignalP 5.0 improves signal peptide predictions using deep neural networks. *Nat. Biotechnol.* **37**, 420–423 (2019). 30778233 10.1038/s41587-019-0036-z

[14] Kall, L., Krogh, A. & Sonnhammer, E. L. Advantages of combined transmembrane topology and signal peptide prediction–the Phobius web server. *Nucleic Acids Res.* **35**, W429–432 (2007). 17483518 10.1093/nar/gkm256 PMC1933244

[15] Krogh, A., Larsson, B., von Heijne, G. & Sonnhammer, E. L. Predicting transmembrane protein topology with a hidden Markov model: application to complete genomes. *J. Mol. Biol.* **305**, 567–580 (2001). 11152613 10.1006/jmbi.2000.4315

[16] Sperschneider, J., Dodds, P. N., Singh, K. B. & Taylor, J. M. ApoplastP: prediction of effectors and plant proteins in the apoplast using machine learning. *New Phytol.* **217**, 1764–1778 (2018). 29243824 10.1111/nph.14946

[17] Sperschneider, J. *et al*. LOCALIZER: subcellular localization prediction of both plant and effector proteins in the plant cell. *Scientific Reports* **7**, 44598 (2017). 28300209 10.1038/srep44598 PMC5353544

[18] Sperschneider, J., Dodds, P. N., Gardiner, D. M., Singh, K. B. & Taylor, J. M. Improved prediction of fungal effector proteins from secretomes with EffectorP 2.0. *Mol. Plant Pathol.* **19**, 2094–2110 (2018). 29569316 10.1111/mpp.12682 PMC6638006

[19] Yu, G. C., Wang, L. G., Han, Y. Y. & He, Q. Y. clusterProfiler: an R Package for comparing biological themes among gene clusters. *Omics-a Journal of Integrative Biology* **16**, 284–287 (2012). 22455463 10.1089/omi.2011.0118 PMC3339379

[20] Wang, Y. P. *et al*. MCScanX: a toolkit for detection and evolutionary analysis of gene synteny and collinearity. *Nucleic Acids Res.* **40**, e49 (2012). 22217600 10.1093/nar/gkr1293 PMC3326336

[21] Hu, K. *et al*. HiTE: a fast and accurate dynamic boundary adjustment approach for full-length transposable element detection and annotation. *Nat. Commun.* **15**, 5573 (2024). 38956036 10.1038/s41467-024-49912-8 PMC11219922

[22] Marçais, G. *et al*. MUMmer4: A fast and versatile genome alignment system. *PLoS Comput. Biol.* **14**, 14 (2018). 10.1371/journal.pcbi.1005944 PMC5802927 29373581

[23] Goel, M., Sun, H. Q., Jiao, W. B. & Schneeberger, K. SyRI: finding genomic rearrangements and local sequence differences from whole-genome assemblies. *Genome Biol.* **20**, 13 (2019). 31842948 10.1186/s13059-019-1911-0 PMC6913012

[24] Goel, M. & Schneeberger, K. plotsr: visualizing structural similarities and rearrangements between multiple genomes. *Bioinformatics* **38**, 5328–5328 (2022). 35561173 10.1093/bioinformatics/btac196 PMC9113368

[25] *NCBI Sequence Read Archive* https://identifiers.org/ncbi/insdc.sra:SRP546896 (2024).

[26] *NCBI Sequence Read Archive* https://identifiers.org/ncbi/insdc.sra:SRP312258 (2021).

[27] *NCBI GenBank* https://identifiers.org/ncbi/insdc.gca:GCA_049000075.1 (2025).

[28] *NCBI GenBank* https://identifiers.org/ncbi/insdc.gca:GCA_048999985.1 (2025).

[29] Zeng, Q. D. Haplotype-resolved T2T genome assembly of the dikaryotic wheat fungus *Rhizoctonia cerealis*, *Figshare*10.6084/m9.figshare.28112558 (2024).

[30] Simao, F. A., Waterhouse, R. M., Ioannidis, P., Kriventseva, E. V. & Zdobnov, E. BUSCO: assessing genome assembly and annotation completeness with single-copy orthologs. *Bioinformatics* **31**, 3210–3212 (2015). 26059717 10.1093/bioinformatics/btv351

[31] Okonechnikov, K., Conesa, A. & García-Alcalde, F. Qualimap 2: advanced multi-sample quality control for high-throughput sequencing data. *Bioinformatics* **32**, 292–294 (2016). 26428292 10.1093/bioinformatics/btv566 PMC4708105

[32] Yu, H. Y., Shi, C. M., He, W. M., Li, F. & Ouyang, B. PanDepth, an ultrafast and efficient genomic tool for coverage calculation. *Briefings in Bioinformatics* **25**, 256–258 (2024). 10.1093/bib/bbae197 PMC11066894 38701418

[33] Li, K. P., Xu, P., Wang, J. P., Yi, X. & Jiao, Y. N. Identification of errors in draft genome assemblies at single-nucleotide resolution for quality assessment and improvement. *Nat. Commun.* **14**, 12 (2023). 37848433 10.1038/s41467-023-42336-w PMC10582259

[34] Rhie, A., Walenz, B. P., Koren, S. & Phillippy, A. M. Merqury: reference-free quality, completeness, and phasing assessment for genome assemblies. *Genome Biol.* **21**, 27 (2020). 32928274 10.1186/s13059-020-02134-9 PMC7488777

[35] Ou, S. J., Chen, J. F. & Jiang, N. Assessing genome assembly quality using the LTR Assembly Index (LAI). *Nucleic Acids Res.* **46**, 11 (2018). 30107434 10.1093/nar/gky730 PMC6265445

[36] Wang, Y. *et al*. shinyCircos-V2.0: Leveraging the creation of Circos plot with enhanced usability and advanced features. *Imeta* **2**, e109 (2023). 38868422 10.1002/imt2.109 PMC10989951

